# Fertility and contraceptive decision-making and support for HIV infected individuals: client and provider experiences and perceptions at two HIV clinics in Uganda

**DOI:** 10.1186/1471-2458-13-98

**Published:** 2013-02-02

**Authors:** Rhoda K Wanyenze, Glenn J Wagner, Nazarius M Tumwesigye, Maria Nannyonga, Fred Wabwire-Mangen, Moses R Kamya

**Affiliations:** 1 Makerere University School of Public Health, Kampala, Uganda; 2 RAND Corporation, Santa Monica, CA, USA; 3 Nsambya Home Care HIV Clinic, Kampala, Uganda; 4 Makerere University School of Medicine, Kampala, Uganda

**Keywords:** Family planning, Fertility, HIV, Contraception

## Abstract

**Background:**

Some people living with HIV/AIDS (PLHIV) want to have children while others want to prevent pregnancies; this calls for comprehensive services to address both needs. This study explored decisions to have or not to have children and contraceptive preferences among PLHIV at two clinics in Uganda.

**Methods:**

This was a qualitative cross-sectional study. We conducted seventeen focus group discussions and 14 in-depth interviews with sexually active adult men and women and adolescent girls and boys, and eight key informant interviews with providers. Overall, 106 individuals participated in the interviews; including 84 clients through focus group discussions. Qualitative latent content analysis technique was used, guided by key study questions and objectives. A coding system was developed before the transcripts were examined. Codes were grouped into categories and then themes and subthemes further identified.

**Results:**

In terms of contraceptive preferences, clients had a wide range of preferences; whereas some did not like condoms, pills and injectables, others preferred these methods. Fears of complications were raised mainly about pills and injectables while cost of the methods was a major issue for the injectables, implants and intrauterine devices. Other than HIV sero-discordance and ill health (which was cited as transient), the decision to have children or not was largely influenced by socio-cultural factors. All adult men, women and adolescents noted the need to have children, preferably more than one. The major reasons for wanting more children for those who already had some were; the sex of the children (wanting to have both girls and boys and especially boys), desire for large families, pressure from family, and getting new partners. Providers were supportive of the decision to have children, especially for those who did not have any child at all, but some clients cited negative experiences with providers and information gaps for those who wanted to have children.

**Conclusions:**

These findings show the need to expand family planning services for PLHIV to provide more contraceptive options and information as well as expand support for those who want to have children.

## Background

As HIV care and treatment access continues to expand, people living with HIV/AIDS (PLHIV) are living longer and healthier lives. For the PLHIV whose health has been restored their concerns are shifting from ill health and medications to achieving full integration into communities and living a productive life, including having children [1,2]. In Africa, studies have documented societal expectations in relation to childbearing, specifically, pressures to have children, the need to have boys as heirs, and large families [3,4]. Such expectations and pressures also influence the fertility desires among PLHIV [5,6]. Whereas several studies show that PLHIV are more likely not to want to have more children once diagnosed with HIV, literature also shows that a substantial proportion (20-50%) of men and women living with HIV desire to have children [7,8].

Family planning (FP) support and decisions for HIV infected individuals have been a major focus of HIV prevention interventions, especially prevention of mother to child HIV transmission [9]. Prevention of unplanned pregnancies among PLHIV is the second prong among the recommended PMTCT approaches and scale up of these interventions has been ongoing over the last decade [10]. However, the scale-up of FP for PLHIV has largely focused on availability of contraceptives, and has not comprehensively addressed their fertility decisions and desires [11]. Focusing on only preventing unplanned pregnancies may lead to increased risk of HIV transmission to sexual partners and children, for PLHIV who attempt to have children without adequate information and support. Furthermore, PLHIV who want to have children but perceive that such desires are stigmatized by their providers and thus cannot turn to their providers for support, may be vulnerable to discontinuing HIV care, having children unsafely, and increasing risk of transmission to their partners and unborn children [12]. Thus, providers should offer comprehensive support to meet the needs of both the PLHIV who want to have children and those who are sexually active and wish to prevent pregnancy [13]. However, this balance is not always achieved for various reasons including health system challenges, provider attitude and stigma [6,12,14].

This paper broadly explores the FP decisions among HIV infected men and women at two HIV clinics in Uganda, including their decisions to have or not to have a child and the support that is availed to them as well as their contraceptive preferences, fears and challenges. The two clinics had integrated FP services but one offered information and contraceptives on site while the other offered only information, with informal referral for contraceptives elsewhere.

## Methods

This was a descriptive cross-sectional study, using qualitative data collection methods, including focus group discussions, in-depth interviews and key informant interviews. The qualitative interviews were conducted during February to June 2011, as part of a larger study to assess family planning practices among HIV infected clients at the two HIV clinics in Kampala, Uganda.

### Study sites

The study was conducted at Mulago Immune Suppression Syndrome (ISS/HIV) clinic located within Mulago national referral and teaching hospital in Kampala, and Nsambya Home Care (NHC), a Catholic founded faith-based clinic/department of Nsambya hospital in Kampala. Mulago had fully integrated FP including onsite delivery of contraceptives whereas NHC only provided information with no onsite provision of contraceptives. Both Mulago and NHC had over 9,000 registered HIV clients in care. However, NHC had a prominent community component including visits to client homes, while Mulago predominantly provided facility-based care. NHC also provided HIV care and treatment for children and adolescents while the Mulago HIV clinic provided only adult care. NHC was deliberately included because we anticipated that the patients in this clinic may be more disadvantaged in terms of accessing family planning services. Thus, we wanted to explore their experiences and challenges as well as coping strategies.

### Data collection methods

We conducted focus group discussions and in-depth interviews with patients at both clinics. Interviews were conducted with adult men and women and adolescent boys and girls (15-19 years) in order to get the experiences of these distinct categories of PLHIV. Additionally, key informant interviews were conducted with HIV care providers (including clinicians, nurses, and counselors) and clinic administrators. We conducted FGDs in order to obtain the broader views regarding participant (patient) experiences with contraceptives, their contraceptive preferences as well as fertility desires. Having obtained the big picture from FGDs, we then conducted IDIs with the PLHIV to explore their individual ‘lived’ experiences. Interviews with providers assessed their views and support provided to the patients. Overall, 106 participants were interviewed, including 84 through FGDs, 14 in-depth interviews, and 8 key informant interviews. The 84 FGD participants included 12 adolescent boys, 15 adolescent girls, 25 adult males and 32 adult females. The providers interviewed included two clinic managers, two doctors, and four nurses/counselors. The number and category of interviews conducted are summarized in Table 1.

**Table 1:** Category and number of interviews conducted

**Methods**	**Category of participants**	**Number of Interviews**	**Total number of participants**
Focus Group Discussions	Adolescent males	2 groups all at NHC	12
	Adolescent females	2 groups all at NHC	15
	Adult males	4 groups (2 at NHC and 2 at Mulago)	25
	Adult females	5 groups (2 at NHC and 3 at Mulago)	32
In-depth interviews	Clients (both men and women)	14 IDI (8 at NHC and 6 at Mulago)	14
Key Informant Interviews	Clinic managers (one at each site), clinicians (one at each site), counselors/nurses (two at each site)	8 KIIs (4 at each clinic)	8
			106 (including 84 from FGDs)

We purposively selected sexually active men and women (those who reported that they had sex within 12 months), aged 15-49 years (within the reproductive age group) and had attended the clinics for at least six months. We included duration of attendance at the clinic, since some of the questions were related to the services delivered at the clinics. Sexually active adolescents were included since the adolescents may encounter different challenges from those of the adults in access to FP services. At each study site, a research assistant with the help of a provider, non-systematically selected participants from the outpatients waiting to be seen by clinicians. The provider explained to all patients about the study related to FP needs of PLHIV, before the selection of potential participants. A brief screening tool was used to assess eligibility of selected participants (age, sexually active, duration of enrollment). Selection of providers was done with the help of clinic management; the clinic managers were requested to identify providers who were involved in counseling or provision of FP services at the clinic. The interviewers then approached each of the selected providers to schedule and conduct the interviews. All the FGDs and IDIs were held in the local language (Luganda) while key informant interviews were conducted in English.

Interview guides were developed for the focus group discussions, in-depth individual interviews and key informant interviews. Open-ended questions were used to allow for exploring new leads and generating rich personal narratives, and prompts were used when needed. The key questions or areas of focus included: What are their contraceptive preferences and are they able to access these methods? What challenges, if any, do they experience in accessing contraceptives? For NHC where contraceptives were not available, are the PLHIV able to access supplies at other sites? What influences the decisions to have/not to have children? What are the influences of HIV status (individual and partner HIV status) on fertility desires? What support do they get from providers when they want to have children?

### Ethical issues

Participants provided verbal informed consent, following explanation of the purpose of the study and procedures. Interviewers read to them pre-designed information about the study and documented the participants’ agreement to be interviewed. The participants were assured of anonymity and confidentiality of the data; the interviews were conducted in a private environment and the transcripts did not bear the participant names. The sexually active adolescents (15-19 years) were handled as ‘mature’ minors, with waiver of parental consent. The Uganda National Council for Science and Technology (UNCST) defines children (mature and emancipated minors) who may independently provide informed consent to participate in research. Mature minors are individuals 14-17 years of age who have drug or alcohol dependency or a sexually transmitted infection; while emancipated minors are those who are pregnant, married, have a child or cater for their own livelihood [15]. Obtaining consent from the parents or guardians of sexually active HIV infected adolescents may expose them to risks of stigma, discrimination or other such harms from parents/guardians who may otherwise not know that they are sexually active or HIV infected. Because of these considerations, adolescents provided consent but were given the option of involving their parents and/or guardians in the consent process. They were thus treated as ‘mature minors’. The participants who were clients at the clinics were given 5,000 Uganda shillings (equivalent to 2.5 USD at the time) as compensation for their time while providers received 10,000 shillings. The study was approved by Makerere University School of Public Health Higher Degrees Research and ethics committee, and the UNCST.

### Data management and analysis

All interviews were digitally recorded and a designated note taker was present in all groups and individual interviews -- the notes augmented the recording. Experienced research assistants directly transcribed and translated the recordings from Luganda into English. Based on the key questions and study objectives, a coding system was developed before the transcripts were examined. The initial step in the analysis was to read through all the transcripts several times while making notes in the transcript. The notes were reviewed by multiple readers and themes identified and coded, to ensure that all the key themes were captured. The research assistants and investigators used an empirical approach to modify the pre-determined themes. After verifying and reviewing the coded and labeled responses, the authors identified major themes. Open coding was done and the codes were grouped into categories in order for themes to be identified (as stipulated by the methods of Graneheim & Lundman, 2004 [16]. Codes were grouped into categories and then themes and subthemes further identified. This was intended to indentify similarities as well as differences in experiences and opinions across various categories of participants (men, women, adolescents, providers, and the two clinics). Qualitative latent content analysis technique was used. The data were therefore condensed without losing quality.

## Results

All the respondents both providers and clients appreciated the role of FP and emphasized the need to integrate FP into HIV services. Providers highlighted the need to have more comprehensive reproductive health services for PLHIV, including cervical cancer screening for HIV infected women. Adolescents (both girls and boys) appreciated the need for FP but thought the adults needed it more since they were having too many children. Participants noted that the education on family planning needs to be broadened beyond the current focus on using contraceptives only, to include planning for and spacing of children as well as having the number of children that one can afford to care for.

*‘We are 11 children in our family, and our parents left us. I don’t live in good conditions. Family planning would have helped a lot’*. Male Adolescent NHC

### Preferred contraceptive methods and access

Most IDI and FGD participants, adolescents, adult men and women, preferred condoms. They noted that the clinics emphasized condoms to prevent HIV re-infection and unplanned pregnancies. They also noted that the condoms have limited side effects and can prevent HIV re-infection or transmission of sexually transmitted infections. ‘*Some men fear to disclose their HIV status to their women but find it easy to use condoms under the pretext that it is for family planning*’, said one adult male FGD participant. Some providers however felt that the condoms were largely marketed for prevention of HIV and not as a method of family planning ‘*The message on condom use has been packaged to emphasize prevention of STDs and HIV but we have not gone ahead to emphasize it as a method of FP*’ Female provider NHC. Male condoms were the most preferred method among both men and women ‘*they are easily accessible, cheap and they are easy to use*’, said an adult male at Mulago. However, some women and men said condoms reduce sexual pleasure.

Some women cited challenges with asking their partners to use condoms and as such preferred methods that they could use without telling their partners or asking for their permission (e.g. injectables; IUDs). They however noted that the intrauterine devices and implants were not easily accessible and were expensive. Some men also noted that the IUDs are good and have no side effects but said most people have not had an opportunity to be educated about them. ‘*My wife was wondering how a ring that is inserted in her body can prevent one from getting pregnant. What if this ring disappears in her body or gets into her blood stream…?*’ said one adult man at Mulago.

Both adult and adolescent women mentioned pills but had concerns about taking additional pills. Despite this concern, some women said they liked pills ‘*because they enable one to enjoy sex without reducing the pleasure like a condom*’ said one adult woman at Mulago. Adolescent girls had concerns about pills and injectables, ‘*Pills and injections can prevent women from having children in the future*’ Female adolescent NHC. Some women said they had experienced several side effects with pills and injectables. Men also said pills and especially injectables have side effects including abdominal complications, prolonged periods, infertility and child abnormalities and noted that injections cause weight problems, high blood pressure, heart palpitations, and sleeplessness. However, some women who had injectables said they did not have major issues with them and thought they were better since they did not need to use them daily like pills. They however noted that the cost of the injectables had increased and was prohibitive.

*‘Most women use injections because you do not need to be on tension like swallowing pills. But injections have become expensive. It used to be about 2500/= [1.25USD] then increased to 3500/= [1.75USD] and as we talk now an injection costs 5000/= [2.5USD]. This is expensive and some of us may not afford*.’ Adult female NHC

Some men said they had heard about vasectomy but had mixed feelings about it and were not aware of anyone who had done it. “*… men have a fear that this could lead to their inability to have sex*” said one adult man at Mulago. Men also noted that providers focus mostly on the women and ask them to bring their wives when they ask FP related questions. ‘*As men we should also be given this information, not to say that every time you want to know about family planning you should bring your wife first’*. Adult male Mulago

Unlike the adults, adolescents mentioned abstinence as a method of preventing pregnancy. All adolescent interviews highlighted challenges with accessing FP information. They said that they attended workshops organized by counselors but FP issues were not discussed. They noted that some of them would want to use contraceptives but they are not aware of the options and the advantages and disadvantages of the different types of contraceptives. They noted that some of their colleagues were sexually active but feared to tell the counselors.

*‘…the counsellors see us as young and rarely tell us about family planning, we only see posters in their office*’, said one female adolescent at NHC.

#### Challenges with accessing services at the clinics and other facilities

Respondents from NHC mentioned ‘moon beads’ a rhythm method that is used to educate women at their clinic. Some clients however felt that the moon beads were not very reliable.

*‘…most people do not know how to use these beads; seasons change, people’s lives change and if they go by these moon beads they find themselves pregnant and when they come back here in tears healthcare workers may not believe them*’. Adult man NHC

Some women said the FP information was not given frequently enough. ‘*It is only counselors who ask us about FP every time we go to their rooms*’, said one adult female at NHC*.* The respondents at NHC said they were given information on contraceptive use and referred to get the supplies from other facilities. However, they noted that some people did not go to the facilities they were referred to. ‘*I wish they could also give us the moon beads to take home. They use them for teaching but we cannot own them*’. Female adult IDI NHC. Some clients appreciated the HIV services and felt it was their responsibility to get the FP services elsewhere. However, women noted challenges with using private and other facilities for FP services.

*‘…we use services of the private clinics or doctors who are not aware of our medical condition*. …*you cannot go telling everybody that you are positive; they may give us a drug or injection which conflicts with the ARVs we are taking*’, said one adult woman at NHC.

Other respondents, especially adolescents, noted that the cost of the contraceptives is high.

*‘In other clinics family planning services are expensive so it can only be accessed by those who have money*’. Female adolescent NHC.

The Mulago respondents noted that the information was freely provided and supplies were available although a few cited stock out of condoms occasionally. They also noted that sometimes the health workers are very busy, with other activities or the clients are many, and they do not want to wait for contraceptives so they leave after getting other drugs.

*‘The quality of family planning is good if you are patient. I do not have the patience to wait to see the family planning person, but I have not had any other challenge*’. Female adult IDI Mulago

### Decisions to have children

All interviews for adults and adolescents reveled that many PLHIV desired to have children. ‘*We are living with HIV but we love children, we want to have more…*’, said an adult female at NHC. The decision to have or not to have children was influenced by several factors including having few children or none; composition of the children (having only boys or only girls); getting into a new sexual relationship; pressures from family members and community and the HIV status of the sexual partner. Respondents (adolescents and adults) noted the need to have children in order to be accepted and to ‘*please our parents*’ as one male adolescent said. The desire to have at least one child was raised in all adolescent interviews. Adolescents expressed the desire to have children in future and a fear of dying without having a child.

*‘If a person passes on without a child, you are taken as a person who has lived a meaningless life. In Buganda, it is like a taboo*’*.* Adolescent male NHC

#### The desire to have more children for PLHIV who already have children

Respondents across all categories felt that it was not good to have only one child. They felt this was unfair to the child and that every child needs to have a brother or sister.

*‘Personally I was diagnosed HIV positive before I had any child, we have so far had one child and I would like to have 5 even though I am HIV positive*’. Adult male Mulago

They noted that those who have HIV infected children try to have more children in an attempt to get an HIV free child. Some respondents also said their partners may want to have more children because they are not aware of their HIV positive status. Ability to care for more children was cited frequently as influencing the decision to have more children.

Respondents expressed the need to have a male child so that they can have an heir. ‘*My two children are girls, so I have no one to inherit my property when I die*’ said one adult man at Mulago. However, respondents who had only boys also expressed the need to have girls.

*‘I have two boys, one is 15 years and the other is 9 years old but I would like to have some girls; I want to have 4 children in total*’. Adult female IDI NHC

Respondents mentioned cultural pressures to have children and especially large families as a problem. ‘*Even our parents put us on pressure to produce*’, said one adolescent girl at NHC. They cited having large families and having twins as prestigious. They also said men want to have boys.

*‘A man might want a boy. Then they produce 12 children, still looking for a boy*’ said one female adolescent during a FGD at NHC. ‘*They say that the happiness of a parent comes from having many children*’*, said another* adolescent girl at NHC*.*

Respondents noted the need to have a child in order to strengthen and maintain their relationships. They mentioned that even those who already have children may be forced to have more when they get a new sexual partner.

*‘… Imagine a situation where you have a man who is taking care of you but you have not produced with him. To keep the relationship going and strong, I will be forced to produce so that I do not lose the man*’. Adult woman, FGD Mulago

Respondents also said they wanted to have children so that they can have someone to care for them later in life. ‘*I wish to have children for security when am very old or sick*’. Adult female NHC*.* However, some adolescents said the pregnancies are sometimes accidental.

#### Decisions not to have children

Several clients noted that it is easier to decide to have children when their partners are also HIV infected. They said they worried about surviving in order to look after their children but felt this was less stressful than concerns about infecting the other partner.

*‘My wife is negative and I am positive. We have 2 boys and she wants to have a girl. Every time my wife says that we should have another child, I tell her I am sorry I can’t do that. Who will take care of the children when we are all gone, if I infect her? IVF would be the way to go but I hear it is very expensive …*’. Adult male Mulago.

Respondents also noted that people that already have several children may not want to have more when they learn their HIV positive status.

*‘I had 5 children when I learnt I was HIV positive. I do not want to have any more children. People living with HIV should be sensitized not to have any more children but those who are childless should be helped to have children without infecting them*’. Adult male, FGD Mulago

They felt that having many children when one is sick could be a burden. Most respondents raised ability to care for the children as very important and cited various costs such as feeding, clothing and school fees. Others cited ill health as influencing the decision not to have children. However, they noted that this may change when they improve after getting into care or starting treatment.

*‘…we all went through that stage but this usually changes as time goes by and we become stronger. I spent 4 years thinking I would not have any more children. But with time one gains courage and they decide to have children*’. Adult male NHC

#### Information and support given by providers on contraceptive use and childbearing

Clients noted that counselors talked about contraceptive use and when to have children for those that want to have children. They also talked to them about PMTCT services to ensure that their babies are protected. They noted that this usually happens when they are about to start on ARVs. They said they are advised to use condoms all the time to protect their partners and to avoid pregnancy but those who want to have children are also allowed to have children. ‘*They ask about the CD4 count of the husband and wife. If the CD4 is okay then they can tell you to proceed with having a child*’. Adult female NHC They however noted some challenges for those who want to have children. One adult man noted in a FGD ‘*They ask us whether we want to have children and then emphasize condom use all the time. How do we have these children if we are using condoms all the time?*’. The desire to have more guidance on childbearing issues was also expressed by adult women and adolescents. Adolescents noted that the information is not very comprehensive and does not address all their concerns.

*‘…we are still young and need to bear children. So we need to be well guided on how we can best go about issues of family planning. If am using family planning I need to know when do I stop it if I want to give birth*’, said a female adolescent NHC.

*‘My last born is about 18 years and my husband died a few years ago. I got another man and would like to have children with him. I am 43 years and need guidance on how to conceive and produce a healthy baby without any problem*’. Adult female Mulago

However, another participant from the same clinic said that those who want to have children are asked to talk to the doctors for advice. ‘*They [doctors] allow you one week of not using condoms and after that you resume using them every time*’; Adult man Mulago. One respondent who was not yet on ARVs on the other hand did not seem to be aware of the family planning services. ‘*For me I have not heard about any family planning methods given at this clinic. I think FP is a new thing here*’. Adult man Mulago.

### Attitude and support from health care workers (HCWs) in relation to childbearing

The clients reported that some healthcare providers at the HIV clinics were sympathetic and supportive to clients who wanted to have children. All respondents (IDI and FGDs) from Mulago reported that they are supported when they want to have children.

*‘They support the idea of having children but emphasize that we should seek help here to prevent the baby from being infected*. *They do not discourage us at all*’. Adult female Mulago

However, the voices from NHC were divided. Some said the reception from the providers was good. ‘*If one wants to have a child, you are told to come back and see the counselors; they measure the CD4 count and advise you on how to give birth to a healthy baby*’. Adult female NHC. However, other respondents felt some providers were harsh to clients who want to have children.

*‘I know of a woman who wanted to have an abortion because she feared to come back to the clinic while pregnant*’. Adult male NHC

*‘Health workers do not want to hear that you want to conceive or bear children.’ ‘When we conceive, we have to first hide for some time, about 4 months, because counselors and doctors here do not want us to conceive*’. Adult female NHC

*‘They do not support any one who wants to give birth. This has also forced some women to shy away from treatment due to the fear of how the counselors or doctors will treat them. The time I was pregnant, the doctor shouted at me and scared me that I was going to die, I went back home when my pressure was high and I really suffered*’. Adult female NHC

Unlike the adults, all the adolescents at NHC noted that the counselors were supportive and asked them to be open about their plans to marry and have children.

#### Health workers voices

All the health workers who were interviewed at both clinics said PLHIV have a right to have children and should be helped to do so safely. They felt that clients should consult and be helped when they want to have children and they should have children when they are clinically stable and their CD4 counts are high; they should be taking ARVs well, should attend ANC and be able to deliver at a health facility and receive PMTCT services to ensure the baby is HIV free.

*‘When we had just started giving ARVs we thought they should not get pregnant but over time, I now feel it is okay. When they want to have a baby and they are financially okay and prepared, I think it is okay*’. Female HCW NHC

*‘It is a good idea for them [PLHIV] to have children; but how we implement it is where the gap is. I feel that they should be in regular consultation with their healthcare providers before and during pregnancy up to childbirth*’. Female HCW NHC

Some health workers were however more sympathetic to those clients who had no children at all but felt those who have should not be getting more children.

*‘Some [clients] are careless because you find that someone has HIV and already has 4 children but is going ahead to conceive another child ……. But for a couple who may be newly married and have not had children surely they should give birth but they should consult the counselors and doctors*’*.* Male HCW NHC

*‘It is not bad, but it depends on the type of patient. There are those that already have children. Like someone has 6 children. But then there are those that have 1 and want to have another. We advise them accordingly*’. Female HCW Mulago

Providers noted that those that have fertility problems are referred to the fertility clinic; those that cannot afford the fees at the paying department at NHC are referred to Mulago for free services. NHC providers noted that contraceptives were not available on site ‘*because of our policy as a faith based organization*’; female HCW. Some providers suggested that access to contraceptives be improved through generating a list of centres which provide FP in order to facilitate referral to those that offer free services.

A summary of the patient and provider perspectives is presented in Table 2.

**Table 2:** Contraceptive preferences and childbearing decisions among PLHIV

**Theme**	**Adolescents**	**Adult men and women**
Preferred contraceptive methods	· Largely condoms: easy to use, accessible and do not affect their fertility	· Condoms most preferred: easy to use, cheap and easy to access; limited side effects; prevent pregnancy and HIV transmission (men who fear to disclose their HIV status can use them under the pretext of FP)
	· Some preferred to abstain	· Some women liked injectables, implants: no challenges with remembering to take pills daily, do not like to use or cannot tell partners to use condoms (limit sexual pleasure); can use without telling their partners or asking their permission
	· Fear pills, injectables and other long-term methods because they can prevent them from having children in future	
Challenges/experiences with contraceptives	· Education on FP is limited; providers focusing more on adults	· Intrauterine devices and implants were not easily accessible and were expensive
	· Challenges with accessing FP information; not aware of options and side-effects	· Injectables available but expensive
	· Fear to ask providers for information if providers do not initiate discussion	· Limited education on some methods (e.g. Intrauterine devices; implants)
		· Pills: concerns about pill burden and remembering to take them
		· Side effects with pills and injectables noted by both men and women: abdominal complications, prolonged periods, infertility and child abnormalities; weight problems, high blood pressure, heart palpitations, and sleeplessness
		· Mixed feelings about vasectomy among men
		· Men felt providers focused more on women
		· Providers focused more on PLHIV who had initiated ART
Challenges with accessing contraceptives at the clinics and other facilities	· Cost of the contraceptives high	· Mulago: busy clinic and long waiting time (separate desk/provider for FP)
		· NHC: some of the PLHIV do not go to the facilities where they are referred for contraceptives; challenges disclosing their HIV status to another set of providers; ‘Moon beads’/rhythm method that is talked about at the clinic unreliable
Decisions to have children	· All want to have children; at least one/feared dying without children	· All want to have children; feel it is not good to have one child/unfair to the child
		· Considerations: have few children or none/cultural expectations to have large families; have only boys or only girls; male child to have an heir; getting into a new sexual relationship/to strengthen relationship; pressures from family members and community (to be accepted); HIV status of the sexual partner; ability to care for more children
Decision not to have children	· Health status (transient issue)	· Sero-discordance/concerns about infecting sexual partner
		· Already have several children
		· Health status (transient issue)
Information and support given by providers on childbearing: client perspectives	· Same issues as adults	· Focusing more on contraceptives
		· Not enough attention to child spacing and number of children they want to have
		· Not addressing fertility decisions and support for those who want to have children
Attitude and support from HCWs in relation to childbearing: Client perspectives	· Desired to have more guidance on childbearing	· Providers talk about PMTCT services
	· Counselors were supportive and asked them to be open up about their plans to marry and have children	· Health status: providers emphasized need to have high CD4 count; adherent to ART
		· Noted gaps in information for those who want to have children/told to use condoms all the time and not clear how they can conceive
		· Mulago: all participants felt providers were supportive
		· NHC: divided about support from providers (some felt providers had negative attitude towards childbearing among PLHIV)
Health workers’ voices		· Need to expand SRH services to include cervical cancer screening
		· Support for PLHIV who want to have children not comprehensive enough and needs improvement
		· NHC providers noted gap with not providing FP supplies: suggested formal referral mechanism since their policy does not allow contraceptives on site
		· All felt PLHIV had a right to have children and needed support: need to be clinically stable and have a high CD4 count; should be on ART; should use PMTCT services
		· More sympathetic to those who have no children (e.g. adolescents); those who have children should not get more

## Discussion

Most studies have focused on prevention of unplanned pregnancies among PLHIV, without due attention to the client contraceptive preferences. Studies have also shown high fertility among PLHIV but without highlighting the needs and support provided to PLHIV who want to have children. In this study, we found that the clinics had integrated family planning information and counseling into HIV care and treatment services. However, the information and support was skewed towards contraceptive use in comparison to the support for those who wished to have children. Yet, consistent with other studies, the client interviews revealed the desire to have children among all categories of respondents [17-19].

Information on contraceptive use was provided at both clinics, but some misconceptions and fears still existed about virtually all the methods that were cited, indicating the need to avail a wide range of options, in order to meet the varied preferences. Clients knew about the need to prevent unplanned pregnancies as well as reduce risk of HIV transmission to sexual partners and unborn children. However, with the exception of the condom, use of dual contraceptive and HIV prevention methods was not mentioned in the interviews. Information gaps in relation to contraceptive use were more prominent among the adolescents.

Respondents at NHC highlighted challenges with accessing contraceptives elsewhere, including failure to complete the referral process, challenges with disclosure of HIV status to FP providers at the alternative sites, and cost of contraceptives. Whereas the contraceptives were given free of charge at Mulago the clients at NHC who utilized services at private facilities had to pay for them. These challenges have been cited by several studies and highlight the need for formal referral linkages, in the absence of fully integrated FP services [20,21]. Access to contraceptive supplies and cost of contraceptives as well as factors such as cultural, social and health concerns influence contraceptive use among women and need to be addressed [19].

The findings from this study show an overwhelming desire to have children among adult men, women and adolescents, with all respondents noting that they needed to have children and more than one child at that. The desire to have children was influenced by socio-cultural pressures; pressures to have a child in order to be accepted by family and to have both girls and boys. The family size and gender composition has a strong bearing on the decision to have more children and on the contraceptive choices [22,23]. In this study several respondents cited issues of inheritance and the need to have boys as a key issue in determining fertility [5].

Fertility desires also influenced the contraceptive choices among both adults and adolescents, who selected methods that they felt would preserve their fertility. Whereas providers seemed to consider the number of children, health status (e.g., CD4 count) and adherence to medications by clients and were more sympathetic to PLHIV who had no children at all, the influences of fertility desires among clients go beyond numbers, to issues such as family composition (girls and boys) and new partnerships. Other than ill health and sero-discordance, which related to concerns about risks of transmission to uninfected partners, the reasons for wanting to have children among HIV infected individuals are largely similar to those that have been cited in studies of uninfected individuals [22,23]. Strong social and cultural pressures to have children were prominent in childbearing decisions within our sample, much like other studies of PLHIV in sub-Saharan Africa [5,22,23]. Providers need to expand their understanding and appreciation of such influences in order to provide the necessary support for clients, even where the client decisions may appear to be irrational to providers.

Despite the strong desire by the PLHIV to have children, there were gaps in the support from providers. Clients highlighted the need for more guidance on when and how they can have children safely. The provider views were generally positive and emphasized the need for consultation with other providers who have greater knowledge about safer childbearing. However, some studies have shown fears and challenges around client-provider discussions in relation to fertility [11,12]. These fears are probably influenced by previous negative experiences such as those cited by some clients in this study. Such fears may lead to challenges with retention and adherence to medications for those clients who wish to have children or get pregnant. Whereas providers indicated that their views towards childbearing had changed, some clients still felt otherwise, reflecting a level of perceived stigma from providers.

Safer conception options such as timed intercourse, manual insemination and sperm washing, coupled with ART are becoming increasingly available [13,24,25]. However, only one client cited timed intercourse and providers only mentioned reduced transmission to the child and not the partner, suggesting limited knowledge of such methods among both clients and providers. This is not surprising considering that the FP-HIV integration guidelines at the national level in Uganda do not include provisions for safer conception strategies [26].

We were not able to differentiate between those respondents that had fertility desires and those that had intentions (those that were trying to conceive at the time of the interview). However, the study provides useful insights into the fertility and contraceptive needs of PLHIV.

## Conclusions

In summary, these findings indicate the progress in integration of FP and HIV services at these two clinics but highlight several gaps including providing a wide range of contraceptive options and contraceptive information gaps among adolescents. The study also reveals the need to increase safer conception knowledge among clients and providers, and to improve support for PLHIV who wish to have children.

## Competing interests

The authors declare that they have no competing interests.

## Authors’ contributions

RKW initiated the topic, coordinated the data collection and analysis and wrote the first draft of the paper. GJW, MRK, and NMT made input into the design and review of the paper. MN and FWM contributed to the interpretation and review of the paper. All authors read and approved the final manuscript.

## Pre-publication history

The pre-publication history for this paper can be accessed here:

http://www.biomedcentral.com/1471-2458/13/98/prepub
